# *S*-Acylation controls functional coupling of BK channel pore-forming α-subunits and β1-subunits

**DOI:** 10.1074/jbc.RA119.009065

**Published:** 2019-06-18

**Authors:** Peter J. Duncan, Danlei Bi, Heather McClafferty, Lie Chen, Lijun Tian, Michael J. Shipston

**Affiliations:** Centre for Discovery Brain Sciences, Edinburgh Medical School: Biomedical Sciences, University of Edinburgh, Edinburgh EH8 9XD, United Kingdom

**Keywords:** protein acylation, acyltransferase, protein palmitoylation, potassium channel, ion channel, post-translational modification (PTM), Kcnma1, Kcnmb1, lipid modification, protein trafficking

## Abstract

The properties and physiological function of pore-forming α-subunits of large conductance calcium- and voltage-activated potassium (BK) channels are potently modified by their functional coupling with regulatory subunits in many tissues. However, mechanisms that might control functional coupling are very poorly understood. Here we show that *S*-acylation, a dynamic post-translational lipid modification of proteins, of the intracellular S0–S1 loop of the BK channel pore-forming α-subunit controls functional coupling to regulatory β1-subunits. In HEK293 cells, α-subunits that cannot be *S*-acylated show attenuated cell surface expression, but expression was restored by co-expression with the β1-subunit. However, we also found that nonacylation of the S0–S1 loop reduces functional coupling between α- and β1-subunits by attenuating the β1-subunit-induced left shift in the voltage for half-maximal activation. In mouse vascular smooth muscle cells expressing both α- and β1-subunits, BK channel α-subunits were endogenously *S*-acylated. We further noted that *S*-acylation is significantly reduced in mice with a genetic deletion of the palmitoyl acyltransferase (Zdhhc23) that controls *S*-acylation of the S0–S1 loop. Genetic deletion of Zdhhc23 or broad-spectrum pharmacological inhibition of *S*-acylation attenuated endogenous BK channel currents independently of changes in cell surface expression of the α-subunit. We conclude that functional effects of *S*-acylation on BK channels depend on the presence of β1-subunits. In the absence of β1-subunits, *S*-acylation promotes cell surface expression, whereas in its presence, *S*-acylation controls functional coupling. *S*-Acylation thus provides a mechanism that dynamically regulates the functional coupling with β1-subunits, enabling an additional level of conditional, cell-specific control of ion-channel physiology.

## Introduction

*S-*Acylation, the only dynamic and reversible post-translational lipid modification of proteins, is emerging as a fundamental regulatory mechanism to control signaling protein function in health and disease (1–3). In particular, *S*-acylation has been reported to control multiple aspects of the lifecycle of many ion channels, from assembly to trafficking and functional regulation at the target membrane (4, 5). *S*-Acylation is mediated by a large family of palmitoyl acyl transferases (ZDHHCs) in mammalian cells (6–8). However, for most ion channels ZDHHCs that control ion channel *S*-acylation are largely unknown. Moreover, whether the functional effect of *S*-acylation depends upon the differential assembly of pore-forming and regulatory subunits is poorly understood.

Large conductance calcium- and voltage-activated potassium (BK)^6^ channels play a central role in the control of a diverse array of physiological processes, and disruption of their normal function is associated with a wide variety of disorders ranging from high blood pressure to neurological deficits and metabolic dysfunction (9, 10). The pore-forming α-subunit of BK channels is *S*-acylated at a cluster of cysteine residues in the intracellular S0–S1 loop (11). siRNA knockdown and overexpression studies reveal that *S*-acylation of the S0–S1 loop is controlled by only two of the family of mammalian ZDHHCs, ZDHHC22 and ZDHHC23, with ZDHHC23 playing the major role (12). In recombinant systems, *S*-acylation of the S0–S1 loop is important for robust cell surface expression of the α-subunit, suggesting that loss of S0–S1 *S*-acylation would reduce endogenous BK channel function at the plasma membrane in native systems (11, 12). However, whether *S*-acylation of the S0–S1 loop also controls other aspects of BK channel function or depends upon assembly with regulatory subunits is not known.

To address these issues, we examined the functional effect of *S*-acylation of the S0–S1 loop of the BK channel α-subunit in the presence and absence of the regulatory β1-subunit. In HEK293 cells, co-expression of the regulatory β1-subunit overrides the effect of nonacylation of the α-subunit S0–S1 loop, resulting in a rescue of cell surface expression. However, functional coupling between α- and β1-subunits was significantly attenuated when the α-subunit is nonacylated. In murine vascular smooth muscle cells (VSMCs), the physiological function of BK channels depends on the functional coupling between BK channel α-subunits and regulatory β1-subunits (13–16). Genetic deletion of Zdhhc23 or pharmacological inhibition of *S*-acylation reduced BK channel currents without affecting cell surface expression of BK channel α-subunits. Our studies reveal a novel mechanism to control BK channel physiology through *S*-acylation–dependent control of functional coupling between BK channel pore-forming α-subunits and their regulatory β1-subunits.

## Results

### β1-subunits rescue surface trafficking of S-acylation–deficient BK channel α-subunits

**Figure 1.**
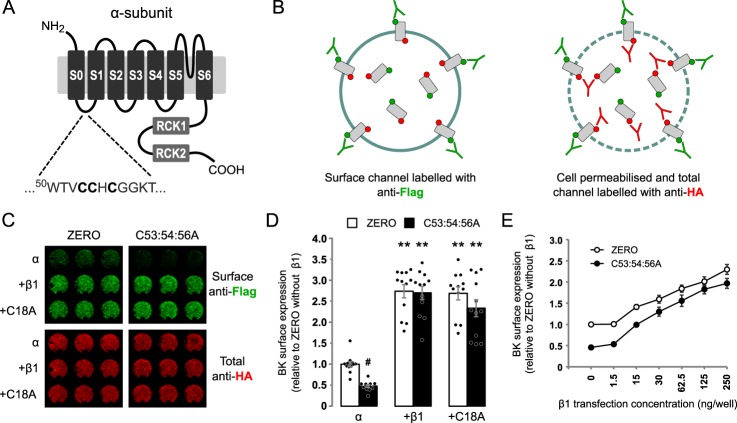
**β1-Subunits override deficit of surface expression of nonacylated α-subunits.** *A*, schematic of BK channel ZERO variant of the α-subunit indicating the cluster of cysteine residues in the S0–S1 intracellular loop *S*-acylated by Zdhhc23 that controls cell surface expression of the α-subunit. *B*, schematic outlining strategy for On-Cell Western assay. *C*, representative On-Cell Western assay of cell surface expression of epitope-tagged BK α-subunit (*ZERO*) or C53:54:56A alone or co-expressed with WT β1 or C18A mutant of β1-subunit in HEK293 cells. Surface BK α-subunit was quantified using an extracellular FLAG-tag (*green*), whereas total BK expression was measured using an intracellular HA tag (*red*) following cell permeabilization. Three replicates from an individual experiment are shown. *D*, quantification of BK channel α-subunit surface expression as a fold of WT α-subunit surface expression alone. There was a significant effect of α-subunit *S*-acylation (*F*_(1,66)_ = 7.090; *p* = 0.0097) and presence of β1 subunits (*F*_(2,66)_ = 120.2; *p* < 0.0001, two-way ANOVA). *E*, effect of increasing concentration of β1-subunit cDNA on cell surface expression of BK α-subunit or C53:54:56A co-expressed with WT β1. All data are means ± S.E. (*n* ≥ 8). ANOVA with Sidak post hoc analysis was used. **, *p* < 0.01 compared with control (α-subunit alone); #, *p* < 0.05 compared with WT (*ZERO*).

The BK channel α-subunit has previously been shown to be *S*-acylated at a cluster of cysteine residues in the intracellular S0–S1 loop (Fig. 1*A*) and is important for robust cell surface expression of α-subunit (11, 12). In a number of systems, β1 accessory subunits have been reported to control trafficking of α-subunits (17–20). We therefore examined the effect of co-expressing β1-subunits on α-subunit surface expression using On-Cell Western assays in HEK293 cells transfected with α- and β1-subunits. In these assays, the α-subunit were labeled with an N-terminal (extracellular) FLAG tag, which was used to measure surface expression in nonpermeabilized cells, and a C-terminal (intracellular) HA tag, which was used to measure total BK α-subunit expression under permeabilized conditions (Fig. 1*B*). There was a significant effect of α-subunit *S*-acylation (*F*_(1,66)_ = 7.090; *p* = 0.0097) and presence of β1 subunits (*F*_(2,66)_ = 120.2; *p* < 0.0001, two-way ANOVA). In these assays, surface expression of the *S*-acylation–deficient BK channel α-subunit mutant C53:54:56A was significantly decreased by ∼50% compared with WT α-subunits when expressed alone in HEK293 cells (Fig. 1, *C* and *D*). Co-expression of the WT β1-subunit increased surface expression of α-subunit ∼3-fold compared with α-subunit alone. Interestingly, surface expression of C53:54:56A was also fully rescued by co-expression with β1-subunits, with surface expression of C53:54:56A in the presence of the β1-subunit not significantly different from the α-subunit in the presence of β1-subunit. To address whether the rescue of cell surface expression of *S*-acylation null α-subunit by β1-subunit simply reflects a saturating response to β1-subunit overexpression, we undertook a dose-response analysis using different levels of β1-subunit. WT β1 subunit dose-dependently increased surface expression of both WT α-subunit and C53:54:56A by similar extents (Fig. 1*E*), suggesting that the presence of β1-subunit overrides the surface expression deficit of C53:54:56A α-subunits independently of saturation or reduced stoichiometry.

To ask whether β1-subunits themselves may be *S*-acylated, we scanned for predicted intracellular *S*-acylated cysteine residues. The prediction suggests that Cys^18^ in the intracellular N-terminal domain of the β1-subunit N terminus is a potential *S*-acylated cysteine residue (Fig. S1*A*). In HEK293 cells, WT β1 incorporates ^3^H-labeled palmitate and mutation of Cys^18^ to alanine (C18A) abolishes incorporation of ^3^H-labeled palmitate (Fig. S1*B*), supporting a role for Cys^18^ as a potential *S*-acylated cysteine. Functionally, mutation of β1 Cys^18^ to alanine (C18A) still promoted surface expression of both α-subunit and C53:54:56A by ∼3-fold, which was not significantly different compared with α-subunit co-expressed with WT β1 (Fig. 1, *C* and *D*). Taken together, this suggest that potential *S*-acylation of the β1-subunit does not play an important role in controlling BK α-subunit surface expression.

### β1 subunit reduces internalization of α-subunit

**Figure 2.**
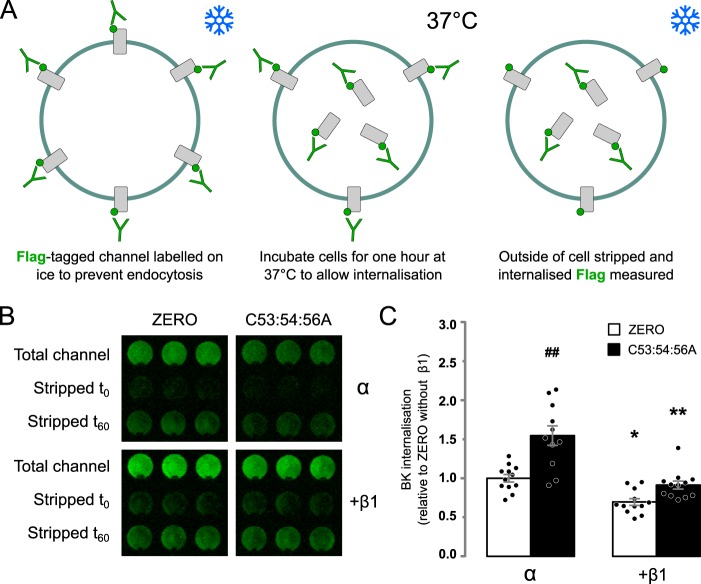
**β1-Subunit retards internalization of α-subunit.** *A*, schematic outlining strategy for internalization assay in HEK293 cells. *B*, representative internalization assay of epitope-tagged BK α-subunit (*ZERO*) or C53:54:56A alone or co-expressed with WT β1-subunit in HEK293 cells. Internalized BK α-subunit was quantified, after 60 min at 37 °C, using the extracellular FLAG tag (*green*) following acid strip of surface staining in nonpermeabilized cells and normalized to surface expression at time 0. Three replicates from an individual experiment are shown. *C*, quantification of BK channel α-subunit internalization relative to the WT α-subunit internalization alone. There was a significant effect of α-subunit *S*-acylation and the presence of β1 subunits (*H*_(3,48)_ = 26.85; *p* < 0.01, Kruskal–Wallis test). All data are means ± S.E. (*n* = 12). Kruskal–Wallis test with Dunn's post hoc analysis was used. *, *p* < 0.05; **, *p* < 0.01 compared with control (α-subunit alone). ##, *p* < 0.01 compared with WT (*ZERO*).

The reduction in surface expression of the nonacylated C53:54:56A α-subunit is, at least in part, a result of reduced forward trafficking, and β1-subunits can promote forward trafficking of α-subunits. To address whether increased channel internalization may also contribute to the decreased C53:54:56A surface expression and whether β1-subunits reduce internalization, we exploited a modified On-Cell Western protocol (Fig. 2*A*). We expressed FLAG-tagged WT or C53:54:56A α-subunit alone or co-expressed with WT β1 in HEK293 cells. BK channels at the cell membrane were labeled using a mouse anti-FLAG antibody, followed by an anti-mouse secondary antibody (IRDye800CW). The cells were then incubated for 1 h at 37 °C to allow channel internalization. The remaining surface staining antibodies were then removed by acid stripping to leave only signal from internalized channels. There was a significant effect of α-subunit *S*-acylation and presence of β1 subunits (*H*_(3,48)_ = 26.85; *p* < 0.01, Kruskal–Wallis test). There was a significantly higher rate of internalization of the C53:54:56A variant (1.55 ± 0.13-fold increase) relative to WT α-subunit (Fig. 2, *B* and *C*). Co-expression of β1 resulted in decreased internalization of both WT and C53:54:56A α-subunit (0.70 ± 0.04 and 0.91 ± 0.05, respectively), suggesting that β1 also acts to increase BK channels at the cell membrane by reducing internalization. Taken together, this suggests that the reduced surface expression of C53:54:56A is due to both a decrease in forward trafficking and an increase in internalization and that β1-subunits in part enhance surface expression by effects on both forward trafficking and internalization.

### S-acylation of the α-subunit S0–S1 loop controls functional coupling with β1 subunit

**Figure 3.**
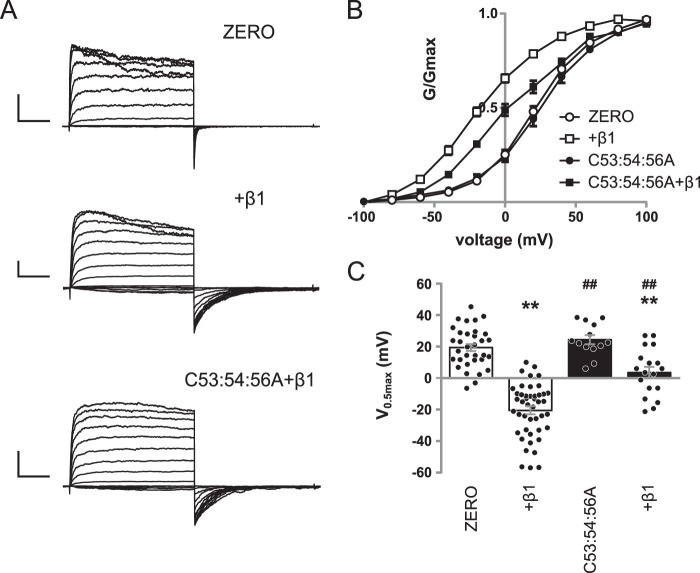
***S*-Acylation of the S0–S1 loop controls functional coupling with β1-subunits.** *A*, representative traces showing outward currents at depolarized potentials from HEK293 cells expressing α-subunit alone (*ZERO*), α-subunit plus β1, *S*-acylation–deficient mutant C53:54:56A plus β1. Outward currents were elicited by 100-ms depolarizing voltage steps from −100 to 100 mV in 20-mV steps, with a holding potential of −120 mV. *Scale bars*, 2 nA/25 ms. *B*, normalized *G*/*G*_max_ conductance voltage relationships for α-subunit (*open circle*), α + β1 (*open square*), C53:54:56A (*filled circle*), and C53:54:56A plus β1 (*filled square*). *C*, summary bar graph showing the corresponding voltage for half-maximal conductance (*V*_0.5max_). There was a significant effect of α-subunit *S*-acylation (*F*_(1,129)_ = 34.28; *p* < 0.0001) and the presence of β1 subunits (*F*_(2,129)_ = 49.24; *p* < 0.0001, two-way ANOVA). All data are means ± S.E. (*n* ≥ 14). **, *p* < 0.01 compared with α-subunit alone; ##, *p* < 0.01 compared with α-subunit in the presence of β1-subunit (ANOVA with Sidak post hoc analysis).

To establish whether *S*-acylation of the S0–S1 loop of α-subunits determines the electrophysiological properties regulated by β1 subunits, we expressed WT α-subunit or *S*-acylation null α-subunit (C53:54:56A) alone or co-expressed with WT β1 with a C-terminal Myc epitope in HEK 293 cells. Macropatch currents were recorded (Fig. 3*A*) from inside-out isolated patches evoked by 100-ms step depolarizations (−100 to 160 mV), and outward current was measured during the sustained phase (80–100 ms into the pulse). There was a significant effect of α-subunit *S*-acylation (*F*_(1,129)_ = 34.28; *p* < 0.0001) and presence of β1 subunits (*F*_(2,129)_ = 49.24; *p* < 0.0001, two-way ANOVA).

Co-expression of α-subunit with β1-subunit (*n* = 48) resulted in robust macropatch currents that displayed a significant left shift of the conductance–voltage (*G*/*V*) relationship compared with α-subunit alone (*n* = 39; Fig. 3, *B* and *C*), which is consistent with previous reports (16). The *V*_0.5max_ of α-subunit alone was 19.5 ± 2.2 mV, whereas the presence of β1 significantly shifted the *G*/*V* relationship to the left to −20.4 ± 2.5 mV. As previously reported (11, 12), the *S*-acylation null mutant C53:54:56A alone (*n* = 17) displayed robust macropatch currents with a *V*_0.5max_ of 24.5 ± 2.9 mV, which was identical to α-subunit alone (19.5 ± 2.2) (Fig. 3, *B* and *C*). However, co-expression of C53:54:56A with saturating levels of β1-subunit resulted in an attenuated left shift of the *G*/*V* relationship compared with α-subunit in the presence of β1-subunit (Fig. 3, *B* and *C*). The *V*_0.5max_ of C53:54:56A in the presence of β1-subunit was 3.6 ± 3.6 mV, which was significantly different from WT α-subunit in the presence of β1-subunit (−20.4 ± 2.5 mV), as well as C53:54:56A alone. This suggests that *S*-acylation may provide a mechanism to dynamically control the functional coupling of α-subunits with β1-subunits.

### Functional coupling is independent of β1 subunit S-acylation

**Figure 4.**
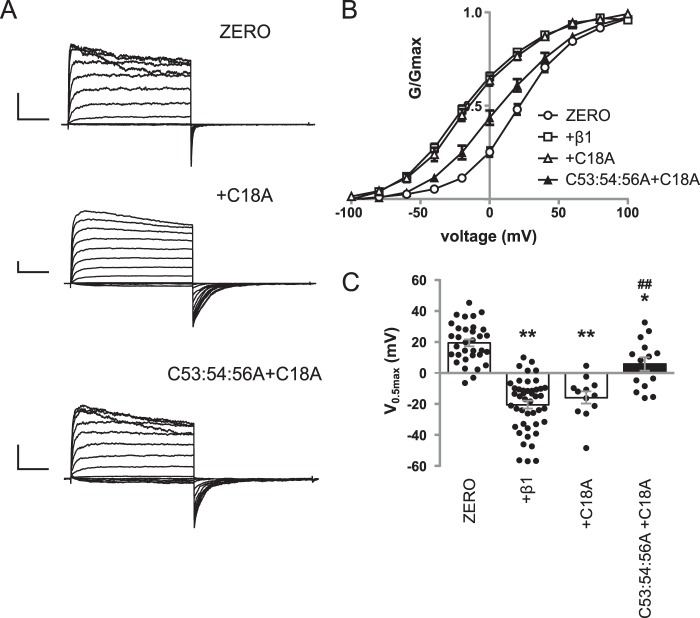
***S*-Acylation of β1-subunit has no effect on functional coupling.** *A*, representative traces showing outward currents at depolarized potentials from HEK293 cells expressing α-subunit alone (*ZERO*), α-subunit plus C18A mutant of the β1-subunit, and *S*-acylation–deficient mutant α-subunit C53:54:56A plus C18A mutant of the β1-subunit. Outward currents were elicited by 100-ms depolarizing voltage steps from −100 to 100 mV in 20-mV steps, with a holding potential of −120 mV. *Scale bars*, 2 nA/25 ms). *B*, normalized *G*/*G*_max_ conductance voltage relationships for α-subunit (*open circle*), α-subunit plus WT β1-subunit (*open square*), α-subunit plus C18A mutant β1-subunit (*open triangle*), and C53:54:56A plus C18A mutant β1-subunit (*filled triangle*). *C*, summary bar graph showing the corresponding voltage for half-maximal conductance (*V*_0.5max_). The data for ZERO and ZERO with WT β1-subunit are controls from Fig. 3 and shown for comparison. There was a significant effect of α-subunit *S*-acylation (*F*_(1,129)_ = 34.28; *p* < 0.0001) and presence of β1 subunits (*F*_(2,129)_ = 49.24; *p* < 0.0001, two-way ANOVA). All data are means ± S.E. (*n* ≥ 14). **, *p* < 0.01 compared with α-subunit alone; ##, *p* < 0.01 compared with α-subunit in the presence of β1-subunit (ANOVA with Sidak post hoc analysis).

To verify that the effect of *S*-acylation on functional coupling is a result of the *S*-acylation status of the α-subunit *per se* and does not involve potential *S*-acylation of the β1 subunit, we expressed WT α-subunit or C53:54:56A alone or pore-forming subunits co-expressed with WT β1 or its corresponding Myc-tagged *S*-acylation–deficient β1 (C18A) mutant subunit in HEK 293 cells (Fig. 4*A*). There was a significant effect of α-subunit *S*-acylation (*F*_(1,129)_ = 34.28; *p* < 0.0001) and presence of β1 subunits (*F*_(2,129)_ = 49.24; *p* < 0.0001, two-way ANOVA). Co-expression of WT α-subunit with C18A β1-subunit (*n* = 14) also resulted in robust macropatch currents and a significant left shift of *V*_0.5max_ to −15.8 ± 3.9 mV compared with α-subunit alone (Fig. 4, *B* and *C*). This left shift in *V*_0.5max_ was not significantly different from that observed with the WT β1-subunit (Fig. 4, *B* and *C*). In addition, C53:54:56A co-expressed with the C18A β1-subunit (*n* = 14) displayed a significantly left-shifted *G*/*V* relationship compared with that of C53:54:56A alone (Fig. 4, *B* and *C*). The *V*_0.5max_ of C53:54:56A in the presence of the C18A mutant β1-subunit was 5.9 ± 4.3 mV, significantly different from that of C53:54:56A but not significantly different from that of C53:54:56A in the presence of WT β1-subunit. Taken together, the data reveal that *S*-acylation of the S0–S1 loop of the α-subunit, but not *S*-acylation of the β1 subunit, is important for the functional coupling between the α- and β1-subunits.

### BK channel α-subunits are S-acylated in VSMCs from mouse aorta

In VSMCs, β1-subunits are highly expressed, and their functional coupling with α-subunits is important for their physiological function (16). Based on our data in HEK293 cells, we predicted that genetic deletion of Zdhhc23 or broad-spectrum pharmacological inhibition of *S*-acylation in VSMC would reduce the outward potassium conductance in VSMCs, which is largely carried by BK channels, without affecting cell surface expression of α-subunits compared with control. siRNA knockdown of ZDHHC23 in HEK293 cells (Fig. S1, *C* and *D*) revealed that this enzyme is not required for *S*-acylation of β1-subunits in contrast to its role in α-subunit S0–S1 loop *S*-acylation. Therefore, any effects of genetic deletion of Zdhhc23 in VSMCs are likely to be due to changes in BK α-subunit palmitoylation rather than β1-regulatory subunits. *S*-Acylated β1-subunit could not be reliably detected in mouse VSMCs (data not shown).

**Figure 5.**
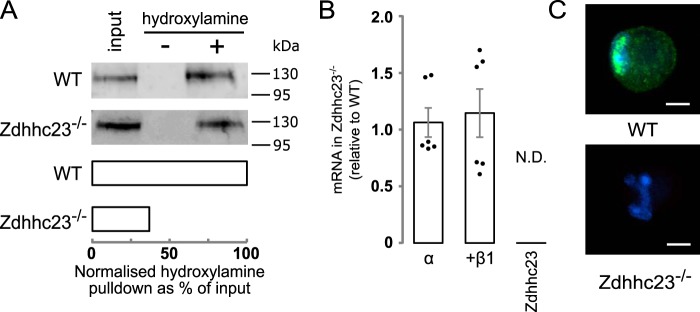
***S*-Acylation of BK channel α-subunit by Zdhhc23 in murine VSMC.** *A*, representative blots and bar chart (*n* = 3) from an acyl-RAC experiment of native BK channel α-subunit *S*-acylation in mouse VSMCs from WT and Zdhhc23^−/−^ mice. *B*, RT-qPCR of BK channel α- or β1-subunit and Zdhhc23 mRNA expression in Zdhhc23^−/−^ VSMCs expressed relative to WT levels (*n* = 6). *C*, representative confocal images of immunocytochemical labeling for Zdhhc23 protein (*green*) and nuclei stained with Hoechst (*blue*) in WT and Zdhhc23^−/−^ VSMCs. *Scale bars* are 5 μm. All data are means ± S.E.

To address whether BK channel α-subunits in mouse VSMCs are endogenously basally *S*-acylated, we performed acyl-RAC on BK channels isolated from mouse aorta VSMCs. *S*-Acylated proteins were captured on thiopropyl-Sepharose beads, run on SDS-PAGE gels, and immunoblotted for BK channel α-subunit. Immunoreactive bands corresponding to the BK channel α-subunit were robustly detected in samples treated with hydroxylamine, which cleaves the thioester linkage exposing reactive cysteines that are subsequently captured by the thiopropyl-Sepharose beads, but not in control salt-treated samples (Fig. 5*A*). To test whether endogenous *S*-acylation of the BK channel α-subunit depended upon Zdhhc23, we first analyzed Zdhhc23 mRNA and protein expression by RT-qPCR and immunocytochemistry, respectively. Acutely isolated aorta VSMCs from WT mice expressed Zdhhc23 at both the mRNA (Fig. 5*B*) and protein (Fig. 5*C*) level, with endogenous Zdhhc23 protein largely expressed in perinuclear and other intracellular compartments consistent with Golgi and ER localization reported in other cell types. In contrast, Zdhhc23 mRNA or protein was undetectable from aorta VSMCs isolated from mice with a global genetic deletion of Zdhhc23 (Zdhhc23^−/−^) (Fig. 5, *B* and *C*). Acyl-RAC performed on VSMCs from Zdhhc23^−/−^ mice displayed a significant (∼60%) reduction in BK channel α-subunit *S*-acylation (Fig. 5*A*). The remaining *S*-acylation is most likely due to lower level of Zdhhc22, which is the only other ZDHHC that can acylate the S0–S1 loop (12). Genetic deletion of Zdhhc23 had no significant effect on BK channel α- or β1-subunit expression in VSMCs (Fig. 5*B*).

### Genetic deletion of Zdhhc23, or global pharmacological inhibition of S-acylation, down-regulates BK currents, independently of changes in cell surface expression in VSMCs

**Figure 6.**
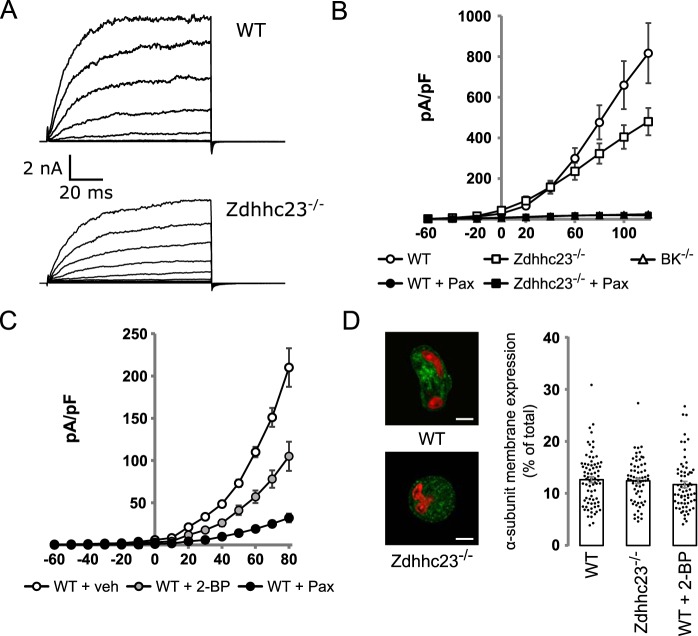
**Genetic and pharmacological inhibition of *S*-acylation reduces BK channel current density in VSMC without affecting cell surface expression.** *A*, representative traces of whole-cell currents from VSMCs isolated from WT (*top panel*) and Zdhhc23^−/−^ mice (*bottom panel*). Outward currents were elicited by 100-ms depolarizing voltage steps from −60 to 120 mV in 20-mV steps, with a holding potential of −80 mV. *B*, current–voltage relationships of the outward steady-state currents expressed as current density (pA/pF) from VSMCs of WT mice (*open circle*; *n* = 18), Zdhhc23^−/−^ (*open square*; *n* = 9) in addition to WT (*filled circle*; *n* = 8) or Zdhhc23^−/−^ (*filled square*; *n* = 7) treated with the specific BK channel blocker paxilline (1 μm) and from VSMCs isolated from BK^−/−^ mice (*open triangle*; *n* = 6). *C*, current–voltage relationships of the outward steady-state currents expressed as current density (pA/pF) from vehicle-treated VSMCs of WT mice (*open circle*; *n* = 24), exposed to 100 μm 2-BP (*gray circles*, *n* = 15) or 1 μm paxilline (*filled circle*, *n* = 18). *D*, confocal images of VSMCs stained for BK α-subunit (*green*) and nuclei stained with TO-PRO3 (*red*). *Scale bars* are 5 μm, with summary bar graph of BK α-subunit plasma membrane expression in VSMCs from WT (*n* = 78) and Zdhhc23^−/−^ mice (*n* = 67), as well as WT VSMCs treated with 100 μm 2-BP (*n* = 69). There were no significant difference between groups (*F*_(2,202)_ = 0.727; *p* = 0.485, one-way ANOVA). All data are means ± S.E.

To test whether inhibition of BK channel *S*-acylation reduces BK currents in VSMC, whole-cell currents were evoked by 100-ms step depolarizations (−60 to 120 mV, holding −80 mV), and outward current was measured during the sustained phase (80–100 ms into pulse) in isolated VSMCs in which intracellular free calcium was buffered to 0.33 μm. Voltage-clamp recordings (Fig. 6, *A* and *B*) revealed large outward currents in WT VSMCs with a current density of 817 ± 148 pA/pF at 120 mV (*n* = 18). This outward current is predominantly carried by BK channels because outward current was reduced by >95% in cells treated with the specific BK channel inhibitor paxilline (1 μm) or from VSMC from BK channel knockout mice (Fig. 6*B*). Outward current density at 120 mV in WT VSMCs pretreated with 1 μm of BK channel blocker paxilline was 21 ± 2 pA/pF (*n* = 8), and that from cells isolated from BK^−/−^ mice was 25 ± 11 pA/pF (*n* = 6) (Fig. 6*B*). VSMCs isolated from Zdhhc23^−/−^ mice showed a significant ∼40% reduction in sustained outward current compared with WT cells at depolarized potentials (Fig. 6*B*). The outward current density at 120 mV was 480 ± 67 pA/pF (*n* = 9) with Zdhhc23 deletion having no significant effect on cell capacitance compared with WT. Cell capacitance in WT was (14.7 ± 1.7 pF) and Zdhhc23^−/−^ cells (12.7 ± 1.6 pF). The reduction in outward current was predominantly through effects on BK current as Zdhhc23^−/−^ cells treated with paxilline had a similar outward current density as paxilline-treated WT cells (Zdhhc23^−/−^ current density in presence of paxilline was 19 ± 4 pA/pF (*n* = 7)). Because genetic deletion of Zdhhc23 did not abolish *S*-acylation of BK channels and only significantly reduced BK currents at depolarized potentials, we tested pharmacological inhibition of *S*-acylation, using the nonselective ZDHHC inhibitor 2-bromopalmitate (2-BP). 2-BP significantly reduced BK currents in WT VSMCs, and the cells were incubated with 2-BP (100 μm) for 12 h prior to recording, with DMSO as vehicle control. 2-BP–treated cells showed a ∼50% decrease in BK current density compared with vehicle control at both negative and positive potentials (Fig. 6*C*). At +20 mV, inhibition was 55.9 ± 4.7% of control, and at −20 mV, it was 52.3 ± 4.3% of control.

To test that inhibition of BK channel α-subunit *S*-acylation would have no effect on surface expression in VSMC, as we predicted from the data in HEK293 cells in the presence of β1-subunit, we quantified surface expression of BK channel α-subunit in native VSMCs by immunocytochemistry and confocal imaging (Fig. 6*D*). Surface expression of BK channel α-subunits, expressed as a percentage of total cellular immunoreactivity, was not significantly reduced (*F*_(2,202)_ = 0.727; *p* = 0.485, one-way ANOVA), compared with controls, in VSMCs from Zdhhc23^−/−^ mice or WT VSMCs treated with 2-BP. BK channel α-subunit surface expression expressed as a percentage of total BK channel expression was 12.6 ± 0.6% in WT cells (*n* = 78), 12.4 ± 0.5% in Zdhhc23^−/−^ cells (*n* = 67), and 11.7 ± 0.6% in 2-BP–treated cells (*n* = 69). Taken together, the data demonstrate that inhibition of BK channel *S*-acylation in VSMCs reduces BK current density independently of changes in surface expression.

## Discussion

In this work we reveal a novel mode of BK channel regulation through *S*-acylation–dependent control of the functional coupling between the pore-forming α-subunits and regulatory β1-subunits. Functional coupling depends upon *S*-acylation of the intracellular S0–S1 loop of the α-subunit rather than the β1-subunit itself. Importantly, *S*-acylation of the S0–S1 loop controls BK channel function in a conditional, context-dependent manner. In the absence of the β1-subunit, *S*-acylation of the S0–S1 loop controls BK channel surface expression through effects on forward trafficking (12), as well as controlling channel internalization from the plasma membrane. However, in the presence of the β1-subunit, this *S*-acylation–dependent control of BK channel α-subunit trafficking is overridden by the β1-subunit, and *S*-acylation of the S0–S1 loop now controls the functional coupling between the α- and β1-subunits. Because S0–S1 loop *S*-acylation can be dynamically controlled by the opposing actions of ZDHHC23 and cognate acylthioesterases (12), this may provide a mechanism to tune functional coupling to cellular needs.

Why would *S*-acylation–dependent dynamic control of functional coupling be important for BK channels in VSMCs? Previous studies in rat and human cerebral arterial smooth muscle cells has revealed an important role for regulation of the calcium sensitivity of plasma membrane localized BK channels through delivery of the β1-subunits to the plasma membrane via phosphorylation-dependent anterograde recycling of β1-subunit–containing endosomes (21). This is important for control of cerebral artery myocyte contractility in particular through nitric oxide signaling pathways. These systems were characterized by a high (>90%) plasma membrane resident population of BK channel α-subunits under basal conditions, with the majority of the β1-subunits being intracellular. However, additional mechanisms are likely required to control functional coupling of BK channels that are already assembled with β1-subunits and resident at the plasma membrane. This might be particularly relevant for cells in which the plasma membrane resident pool of α-subunits is the minority, as in the mouse aortic smooth muscle cells (∼12% total α-subunits resident at the plasma membrane) used in our studies. Thus, *S*-acylation–dependent control of β1-subunit functional coupling in channels in which α- and β1-subunits are already assembled would provide an additional level at which to control BK channel calcium sensitivity and activity.

How might *S*-acylation of the S0–S1 loop control functional coupling? The β1-subunits modulate voltage sensing of BK channels by stabilizing the active configuration of the voltage sensor and thus having large effects on apparent calcium sensitivity (22, 23). Biochemical and lanthanide resonance energy transfer imaging approaches reveal the close association of the transmembrane domains of the β1-subunit with transmembrane domains of the α-subunit (24–26). Moreover, lanthanide resonance energy transfer imaging reveals structural rearrangements in the α-subunit upon interaction with β1-subunits with both the TM1 and TM2 of the β1-subunit close to the S0 and S1 transmembrane segments of the α-subunit in the space between two adjacent voltage sensors (24). Thus, *S*-acylation of the S0–S1 loop may modify the molecular interaction between α- and β1-subunits to control functional coupling. Intriguingly, the cryo-EM structure of the full-length BK channel from *Aplysia californica* reveals that transmembrane domain S0 is tilted with the cytoplasmic aspect of S0 facing the plasma membrane (27). Testing these issues directly will be a significant challenge because complete and specific abolition of S0–S1 loop *S*-acylation by genetic or pharmacological approaches remains technically difficult. Although the site-directed mutagenesis approach is highly useful, as in many other studies of post-translational studies, in this regard we may not be able to completely exclude that some subtle effects on conformation (*e.g.* tilting of the S0 transmembrane domain may be due to the mutations themselves rather than loss of *S*-acylation *per se*.

In many proteins, *S*-acylation of intracellular loop domains plays an important role in stabilizing and controlling the orientation of transmembrane domains in the plasma membrane (1). Whether *S*-acylation of the S0–S1 loop controls S0 orientation to determine functional coupling with β1-subunits remains to be determined. Moreover, whether *S*-acylation of the S0–S1 loop controls other aspects of β1-subunit–mediated regulation of BK channels remains to be determined, for example, the cardiovascular protective effects of omega fatty acids through activation of β1-subunit containing BK channels in the vasculature (28, 29) or the functional coupling between mutant β1-subunits associated with low diastolic blood pressure (30). Pathological changes in BK channel α- and β1-subunit expression and functional coupling are associated with cardiovascular disorders including hypertension, stroke, and atherosclerosis (31). Moreover, functional coupling with β1-subunits is important for physiological control in other systems such as the kidney and bladder (32, 33). Whether dynamic changes in BK channel *S*-acylation and regulation of β1-subunit coupling is important in the physiological control of BK channels in a variety of systems or is disrupted under pathological conditions remains a challenge to be explored. Indeed, more broadly the role of dynamic *S*-acylation of the large number of ion channels now shown to be regulated by *S*-acylation and how zDHHC enzymes themselves are regulated remain major questions in the field (1, 4, 5).

Importantly, the functional effect of *S*-acylation on BK channels is conditional on the presence of the β1-subunit. In the absence of the β1-subunit, *S*-acylation promotes cell surface expression, whereas in the presence of the β1-subunit, *S*-acylation controls functional coupling. Thus, the functional context of *S*-acylation provides an additional, conditional, level of cell-specific control of BK ion channel physiology.

## Experimental procedures

### Reagents

General biochemical reagents used throughout this study were obtained from Sigma–Aldrich and were of analytical-grade quality unless stated otherwise.

### HEK 293 cell culture and transfection

HEK 293 cells were cultured and transfected as previously described (12, 34). The cells were used between passage 18 and 30 and originally obtained from ATCC. HEK293 cells used in this study do not express endogenous the BK α- or β1-subunit as determined by mRNA, protein, or functional assays (11, 12, 19, 34).

The cells were plated on 96-well plates for On-Cell Western assays or on 12-mm coverslips in 6-well plates for electrophysiological experiments, maintained in DMEM containing 10% fetal bovine serum (both Life Technologies), and incubated at 37 °C in 5% CO_2_. The cells were transfected 24 h after plating with the BK channel α-subunit or co-expressed with β1-subunit in a 2:1 ratio of cDNA using Lipofectamine 2000 (Thermo Fisher) or Polyjet (tebu-bio) unless otherwise specified. All expression constructs were previously described (12, 34). The cells were used for experiments 24–48 h post-transfection.

### [^3^H]Palmitic acid incorporation

Incorporation of [^3^H]palmitic acid onto HA-tagged WT β1 subunits or depalmitoylated β1 (C18A) was performed as previously described (4). 48 h after transfection, HEK293 cells were washed with DMEM containing 10 mg/ml fatty acid-free BSA for 30 min at 37 °C, followed by DMEM/BSA containing 0.5 mCi/ml [^3^H]palmitic acid for 4 h at 37 °C. The cells were washed and lysed, and channel fusion proteins were captured using magnetic microbeads coupled to the HA antibody. Proteins were eluted in SDS/PAGE buffer containing 50 mm Tris-Cl (pH 6.8), 5 mm DTT, 1% SDS, 1 mm EDTA, 0.005% bromphenol blue, 10% glycerol, at 95 °C. Recovered samples were separated by SDS/PAGE, transferred to nitrocellulose membranes, and probed for HA (1:1000). A duplicate membrane was exposed to light-sensitive film at −80 °C using a Kodak Biomax transcreen LE (Amersham Biosciences).

### On-Cell Western assays

24 h after transfection, the cells were washed with Hanks' balanced salt solution and trypsinized with 50 μl of trypsin-EDTA. The cells were triturated in 1 ml of GM (DMEM + 10% fetal bovine serum) 20–30 times. A further 1 ml of GM was added, and 100 μl of cell suspension was replated into 96-well plate (Greiner, clear bottomed, black-sided wells, poly-d-lysine–coated). Staining was carried out in quadruplicate for channel detection and in duplicate for cell number (6 wells/transfection). The cells were stained 24 h after replating firstly for the extracellular epitope FLAG and then for the internal C-terminal HA epitope. On ice, mouse anti-FLAG-M2 (Sigma, 1:100 in GM) was applied for 2 h. The cells were washed once in GM and incubated with IRDye800CW goat anti-mouse IgG (Licor, 1:100 in GM) for 1 h on ice. All steps from this point were carried out in the dark. The cells were washed once with GM before fixing in 3.7% formaldehyde solution in PBS for 20 min. Fixative was washed off during permeabilization with five washes of 5 min in PBS + 0.1% (v/v) Triton X-100 before blocking in Odyssey blocking buffer (OBB; Licor) for 1 h. Anti-HA antibody (Immune Systems, 1:1000 in OBB) was applied for 1 h at room temperature and washed off with five washes in PBS + 0.1% TWEEN® 20. Anti-rabbit secondary IRDye690RD (Licor, 1:500 in OBB) was applied for 1 h at room temperature, and for cell number TO-PRO^TM^-3-iodide (1:500) was applied to separate wells. The cells were washed a final five times in PBS + 0.1% TWEEN® 20 before imaging on an Odyssey IR imager with focus offset set to 3 mm, channel intensity 5.0. Images were analyzed using Image Studio Lite Ver5.2 (freeware from Licor). Staining intensity was normalized to cell number and background-subtracted before calculating the ratio of surface FLAG:HA total protein staining. Specificity of FLAG and HA antibodies have been previously demonstrated (11).

### Internalization assay

Transfected HEK293 cells were plated in 96-well plates (Greiner, clear-bottomed, black-sided wells, poly-d-lysine–coated) as for On-Cell Western assays. 24 h after replating, all wells were stained on ice with anti-FLAG-M2 (Sigma, 1:100) for 2 h. The cells were then washed and incubated with IRDye800CW goat anti-mouse IgG (Licor, 1:1000 in GM) for 1 h on ice and protected from light. The cells were then washed, and wells for determination of internalization at time 0 were exposed to ice-cold stripping buffer (0.1 m glycine, 0.1 m NaCl, pH 2.5, in PBS) for 15 min and then washed in growth medium. To determine internalization, labeled and unstripped cells were incubated at 37 °C for 1 h. The cells were then cooled on ice for 5 min before applying ice-cold stripping buffer for 15 min to wells measuring internalization, whereas a subset remained unstripped to measure total channel. All wells were then stained with NucRed^TM^ Live 647 ReadyProbes^TM^ reagent (ThermoFisher) for 30 min on ice. The ;Cells were washed then imaged on an Odyssey IR Imager, focus offset at 3 mm, intensity 5.0 for both 700 and 800 channels. Images were analyzed using Image Studio Lite version 5.2 (freeware from Licor). For each well, staining intensity in the 800 channel was normalized to the NucRed 700 channel signal obtained for each well. Background signal detected in time 0 stripped cells (*i.e.* no internalization) was averaged and subtracted from the other wells. Internalization after 60 min was then normalized to the total surface expression at time 0 to express internalization as a percentage of total BK channel surface expression before internalization.

### Animals

Mice with a genetic deletion of Zdhhc23 (Zdhhc23^−/−^) were generated by KOMP (Zdhhc23^tm1(KOMP)Vlcg^) and subsequently backcrossed for at least 10 generations on a C57/Bl6Ola background before use. Mice with a genetic deletion of the Kcnma1 gene encoding the pore forming α-subunit of BK channels (BK^−/−^) (13) were also generated on the same C57/Bl6Ola background. The mice were caged in groups of two to six under standard laboratory conditions (lights on at 07:00, lights off at 19:00, 21 °C, with tap water and chow available *ad libitum*). WT mice or mice deficient for the zDHHC23 enzyme (Zdhhc23^−/−^) were used from the same litters generated by a cross of mice heterozygous for the *Zdhhc23* allele. The same heterozygous cross-approach was used for BK^−/−^ mice. Tissue collection was performed between 09:30 and 10:30. Animal work was performed with ethical approval from the Animal Welfare and Ethical Review Body of the University of Edinburgh, in accordance with United Kingdom Home Office requirements.

### Vascular smooth muscle cell culture

VSMCs were prepared from male mice aged 2–5 months. Two to four mice were killed by cervical dislocation, and the aortas were removed and stored on ice in a physiological salt solution (Ca^2+^-free PSS) containing 135 mm NaCl, 5 mm KCl, 2.8 mm sodium acetate, 2 mm MgCl_2_, 10 mm HEPES, 10 mm glucose, and 1 mm Na-EGTA (pH 7.3). Smooth muscle and endothelial layers were gently separated from connective tissue and transferred into one of two digest solutions.

For electrophysiology experiments, tissue was transferred to Ca^2+^-free PSS containing 1 mg/ml papain, 0.8 mg/ml DTT, and 0.7 mg/ml BSA and incubated in digest solution for 15 min at 37 °C. Digest solution was removed, and tissue was gently triturated, using a fire-polished glass pipette, in culture PSS containing 130 mm NaCl, 5.9 mm KCl, 2.4 mm CaCl_2_, 1.2 mm MgCl_2_, 10 mm HEPES, 10 mm glucose, and 1% pen-strep. Electrophysiological recordings were obtained from VMSCs up to 6 h post-isolation.

For all other experiments, tissue was transferred to Ca^2+^-free PSS containing 0.8 mg/ml papain, 1 mg/ml DTT, and 1 mg/ml BSA and incubated in digest solution for 30 min at 37 °C. The tissue was transferred to culture PSS and gently triturated. The undigested tissue was transferred to a low-Ca^2+^ PSS (0.05 mm CaCl_2_) containing 1 mg/ml collagenase II, 1 mg/ml hyaluronidase, and 1 mg/ml BSA and incubated for 10 min at 37 °C. The tissue was then transferred to culture PSS and gently triturated. Any undigested tissue was removed, and the two cell suspensions were then combined for experiments.

### Acyl-RAC

Acyl-RAC experiments were based on the protocol as previously described (12, 35). Mice aortas were minced with blades in blocking buffer (100 mm HEPES, 1 mm EDTA, and 2.5% SDS, pH 7.5) on a glass surface. The tissue slurry was then syringed with a 21-gauge needle for 20 strokes and then centrifuged for 5 min at 800 × *g* at 4 °C. The soluble supernatant was then decanted and treated with 0.1% methanethiosulfonate and incubated for 4 h at 40 °C with shaking. Proteins were precipitated in acetone and stored at 20 °C overnight before washing five times in 70% acetone to remove the methanethiosulfonate and allow protein resuspension in 200 μl of binding buffer (100 mm HEPES, 1.0 mm EDTA, 1% SDS, pH 7.5). After removal of 20 μl for input analysis, the remaining blocked proteins were divided into two tubes of 80 μl each and treated with either 0.3 m hydroxylamine (NH_2_OH; Scientific Laboratory Supplies) or 0.3 m NaCl, pH 7.5. Thiopropyl-Sepharose beads were rehydrated in a 1:1 slurry in binding buffer, and 33 μl of beads was added to each tube, incubating for 2.5 h at room temperature. After bead capture, the samples were centrifuged at 13,000 × *g* for 1 min and washed five times in binding buffer. The proteins were then eluted in 33 μl of 2× SDS-LB heated to 60 °C for 10 min.

### RT-qPCR

RNA was extracted from the VSMCs of WT and Zdhhc23^−/−^ mice using the standard protocol from ReliaPrep^TM^ RNA cell miniprep system (Promega). After quantitation by Nanodrop, equal amounts of RNA were reverse-transcribed into cDNA using the Thermoscript^TM^ RT-PCR system (Invitrogen). A non-RT control was prepared in parallel. RNA was primed with an equal 1:1 ratio of random hexamers:oligo(dT) with all incubations carried out on a PCR machine using the following steps: 25 °C for 10 min, 50 °C for 30 min, and 85 °C for 5 min. qPCR was performed on a StepOnePlus real-time PCR system (Applied Biosystems) using Power Sybr Green PCR Mastermix (Applied Biosystems). Approximately 0.7 ng of cDNA was added to each PCR, and all PCRs were performed in triplicate. Endogenous reference control genes were Rn18S and Ipo8, and all primers had been previously validated with efficiencies calculated to be within 0.1 of the control using the equation *e* = 10^(−1/slope)^ − 1. Expression analysis was achieved using the comparative 2^−ΔΔCt^ method. Commercially available RT-qPCR primers were used (Qiagen) except for Kcnma1, Kcnmb1, and Zdhhc23, which were designed and validated in-house with the following sequences: Kcnma1 forward, GTCTCCAATGAAATGTACACAGAATATC; Kcnma1 reverse, CTATCATCAGGAGCTTAAGCTTCACA; Kcnmb1 forward, GCTGTATCACACGGAAGACACTCG; Kcnmb1 reverse, CGCTGGTCTCGTTGACTTGAGG; Zdhhc23 forward, TTGCGAATACATAGATCGAAATGGG; and Zdhhc23 reverse, GCCGAAGTGATTGACAGGTAAG.

### Patch-clamp electrophysiology

Voltage-clamp recordings were obtained from VSMCs in the whole-cell patch-clamp configuration. VSMC suspension was plated directly onto the recording chamber and given 10 min to settle before adding bath solution. The standard bath (extracellular) solution contained 130 mm NaCl, 5.9 mm KCl, 2.4 mm CaCl_2_, 1.2 mm MgCl_2_, 10 mm HEPES, 10 mm glucose, pH 7.4. The standard pipette (intracellular) solution contained 140 mm KCl, 5 mm NaCl, 2 mm MgCl_2_, 10 mm HEPES, 30 mm glucose, 1 mm ATP, 1 mm BAPTA, pH 7.3, with free calcium [Ca^2+^]*_i_* buffered to 1 μm. The free calcium concentrations used in distinct experiments was calculated with MAXCHELATOR.

Macropatch recordings were obtained from transfected HEK293 cells using the inside-out configuration of the patch-clamp technique. All currents were recorded in equimolar potassium gradients. The standard bath (intracellular) solution contained 140 mm KMeSO_3_, 2 mm KCl, 20 mm HEPES, 5 mm HEDTA, pH 7.3, with [Ca^2+^]*_i_* buffered to 10 μm. The standard pipette (extracellular) solution contained 140 mm KMeSO_3_, 2 mm KCl, 20 mm HEPES, 2 mm MgCl_2_, pH 7.3.

All electrophysiological recordings were performed at room temperature and obtained using and Axopatch 200B amplifier and Clampex 10.1 software (both Molecular Devices) with a sampling rate of 10 kHz and filtered at 2 kHz. Patch pipettes were fabricated from borosilicate glass (King Precision Glass, Inc.) using a model P-97 micropipette puller (Sutter Instruments). Pipette tips were heat-polished and had resistances typically between 3–5 MΩ. The data were analyzed using Clampfit 10.1.

### Immunocytochemistry in VSMC

VSMC suspension was cultured on poly-d-lysine–coated glass coverslips for 3 h and fixed with 3.7% paraformaldehyde in PBS for 30 min at room temperature. The cells were washed three times in PBS, permeabilized with 0.3% (v/v) Triton X-100 in PBS for 10 min, and blocked with blocking buffer (BB; 3% BSA in PBS 0.05% Tween) for 1 h at room temperature. The cells were incubated in primary mouse anti-BK antibody (clone L6/60; Antibodies Incorporated, 1:250 in BB) at 4 °C overnight. The cells were washed three times in PBS and incubated in Alexa-488 conjugated anti-mouse secondary antibody (Life Technologies, 1:1000 in BB) for 1 h at room temperature. The cells were washed three times in PBS and incubated in TO-PRO^TM^-3-iodide (Life Technologies, 1:3000 in PBS) for 5 min at room temperature. Alternatively, the cells were stained with rabbit anti-ZDHHC23 (Sigma, 1 in 100) and Alexa 488–conjugated anti-rabbit secondary antibody (Life Technologies, 1 in 1000) with Hoechst nuclear stain (Sigma, 1 in 100) using the same protocol. Finally, the coverslips were washed three times in PBS and rinsed briefly in distilled H_2_O. The coverslips were mounted with Mowiol mounting medium (Calbiochem) containing 1,4-diazabizyclo[2.2.2]octane as anti-fade. The cells were imaged using a Zeiss LSM800 laser scanning confocal microscope equipped with a 63× (NA 1.4) oil immersion objective lens.

BK surface expression was quantified by performing line scans measuring signal intensity through the cell. The BK channel at the cell membrane was quantified by measuring intensity at the periphery and expressed as a percentage of total fluorescence.

### Statistics

The data are expressed as means ± S.E., *n* = number of independent experiments. Statistical analysis was generally performed, as appropriate, by one- or two-way ANOVA with Sidak post hoc multiple comparison tests (GraphPad Prism 6). Significant differences between groups were defined at *p* < 0.05 (*) and *p* < 0.01 (**).

## Author contributions

P. J. D., L. T., and M. J. S. conceptualization; P. J. D., D. B., H. M., L. T., and M. J. S. data curation; P. J. D., D. B., H. M., L. T., and M. J. S. formal analysis; P. J. D., H. M., L. C., L. T., and M. J. S. validation; P. J. D., D. B., H. M., and L. T. investigation; P. J. D. and D. B. visualization; P. J. D., D. B., H. M., and M. J. S. writing-original draft; P. J. D., D. B., H. M., L. C., and M. J. S. writing-review and editing; H. M. and L. T. methodology; L. C. resources; M. J. S. supervision; M. J. S. funding acquisition; M. J. S. project administration.

## Supplementary Material

Supporting Information

## Footnotes

6 The abbreviations used are:

BK: large conductance calcium- and voltage-activated potassium
VSMC: vascular smooth muscle cell
ANOVA: analysis of variance
qPCR: quantitative PCR
2-BP: 2-bromopalmitate
DMEM: Dulbecco's modified Eagle's medium
HA: hemagglutinin
OBB: Odyssey blocking buffer
PSS: physiological salt solution
RAC: resin-assisted capture
HEDTA: *N*-(2-hydroxyethyl)ethylenediaminetriacetic acid
BB: blocking buffer.
