# Supplementary material for: S-Acylation controls functional coupling of BK channel pore-forming α-subunits and β1-subunits
Source: J Biol Chem. 2019 Jun 18;294(32):12066–76. doi: 10.1074/jbc.RA119.009065 (PMC6690687; doi:10.1074/jbc.RA119.009065)
Supplement: Supporting Information [file supp_294_32_12066__index.html]

S-Acylation controls functional coupling of BK channel pore-forming α-subunits and β1-subunits — S-acylation controls functional coupling of BK channel — S-Acylation controls functional coupling of BK channel pore-forming α-subunits and β1-subunits — S-Acylation controls functional coupling of BK channel — Supporting Information 

# *S*-Acylation controls functional coupling of BK channel pore-forming α-subunits and β1-subunits

## Supporting Information

- Supporting Information - Figure S1 - Figure S1
