# Supplementary material for: S-Acylation controls functional coupling of BK channel pore-forming α-subunits and β1-subunits
Source: J Biol Chem. 2019 Jun 18;294(32):12066–76. doi: 10.1074/jbc.RA119.009065 (PMC6690687; doi:10.1074/jbc.RA119.009065)
Supplement: Supporting Information [file supp_RA119.009065_145144_2_supp_348845_pt8ycd.pdf]

SUPPORTING INFORMATION

S-Acylation controls functional coupling of BK channel pore-forming α-subunits and β1-subunits

Peter J Duncan, Danlei Bi, Heather McClafferty, Lie Chen, Lijun Tian & Michael J Shipston

LIST OF MATERIALS

Figure S1

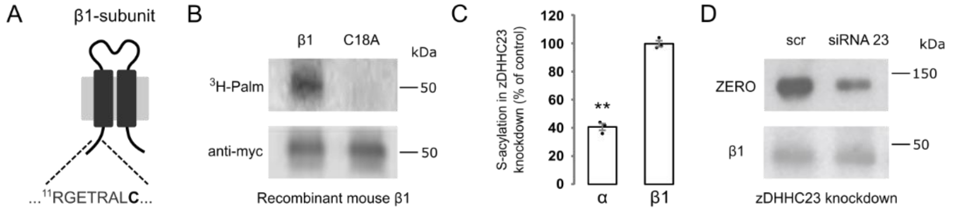

Figure S1: β1-subunit is S-acylated at Cys18 in HEK293 cells

(**A**) Schematic of BK channel β1-subunit indicating cysteine 18 (Cys18) at the intracellular interface of the first transmembrane domain. (**B**) Metabolic incorporation of ^{3}[H]-Palmitate in wild-type β1-subunit but not in C18A mutant β1-subunits expressed in HEK293 cells. (**C**) siRNA knockdown of zDHHC23 reduces S-acylation of α-subunit but not β1-subunit in HEK293 cells. (**D**) Representative blot showing reduced incorporation of ^{3}[H]-Palmitate in α-subunits, but not β1-subunits, of zDHHC23 siRNA-treated cells (siRNA 23) compared to scrambled siRNA (scr). Data are Means ± SE (n = 3), ** $p < 0.01$ compared to scrambled siRNA control (t-test).
